# PLK1 regulation of PCNT cleavage ensures fidelity of centriole separation during mitotic exit

**DOI:** 10.1038/ncomms10076

**Published:** 2015-12-09

**Authors:** Jaeyoun Kim, Kwanwoo Lee, Kunsoo Rhee

**Affiliations:** 1 Department of Biological Sciences, Seoul National University, Seoul 08826, Korea

## Abstract

Centrioles are duplicated and segregated in close link to the cell cycle. During mitosis, daughter centrioles are disengaged and eventually separated from mother centrioles. New daughter centrioles may be generated only after centriole separation. Therefore, centriole separation is considered a licensing step for centriole duplication. It was previously known that separase specifically cleaves pericentrin (PCNT) during mitotic exit. Here we report that PCNT has to be phosphorylated by PLK1 to be a suitable substrate of separase. Phospho-resistant mutants of PCNT are not cleaved by separase and eventually inhibit centriole separation. Furthermore, phospho-mimetic PCNT mutants rescue centriole separation even in the presence of a PLK1 inhibitor. On the basis on these results, we propose that PLK1 phosphorylation is a priming step for separase-mediated cleavage of PCNT and eventually for centriole separation. PLK1 phosphorylation of PCNT provides an additional layer of regulatory mechanism to ensure the fidelity of centriole separation during mitotic exit.

## Abstract (web summary)

Separase-mediated cleavage of pericentrin is an essential step for centriole separation during mitotic exit. Here, Kim *et al.* show that pericentrin has to be phosphorylated by PLK1 in order to be cleaved by separase, which provides an additional layer of regulation for centriole separation during mitotic exit.

## Graphical abstract

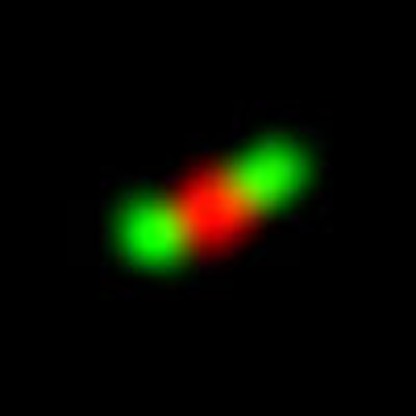

The centrosome is a non-membrane-bound organelle that consists of centrioles surrounded by pericentriolar material (PCM)^1^. Centrioles are duplicated and segregated in a close link to the cell cycle^2^. In the beginning of S phase, a daughter centriole starts to grow next to the mother centriole in a perpendicular angle and remains engaged until mitosis. Once the cells undergo mitosis, daughter centrioles are disengaged and eventually separated from the mother centrioles. A new round of centriole biogenesis is inhibited, as far as the daughter centriole is closely associated to the mother centriole^3^,^4^. Therefore, centriole separation is a licensing step for centriole duplication. A premature separation of centrioles may result in multipolar spindles during mitosis, which is one of the main causes for chromosome aneuploidy^5^.

The centrosome is a major microtubule organizing centre in dividing cells and functions as spindle poles during mitosis. The amount of microtubules emanated from the centrosome is correlated with the amount of PCM, due to the fact that a majority of γ-TuRC is anchored to PCM^6^,^7^,^8^. In fact, a significant amount of PCM becomes accumulated in the centrosome of cells entering mitosis^9^. Defects in PCM accumulation often result in mitotic arrest with monopolar spindles^10^. It is interesting that mutations in PCM protein genes cause congenital brain defects, such as microcephaly^11^. It was proposed that such mutations affect neuronal stem cell division, and lead to reduction of stem cell population and apoptosis^12^.

Pericentrin (PCNT) is a major PCM protein that is involved in mitotic spindle organization, DNA damage checkpoint and primary cilia formation^6^,^13^,^14^. Mutations in *PCNT* gene cause pleiotropic defects, including primordial dwarfism, cancer, mental disorder and ciliopathies^15^,^16^,^17^. Studies with super-resolution microscopy revealed that PCNT spreads out like the spokes of a wheel with the C-terminal domain at the centriole wall^18^,^19^,^20^. PCNT is also crucial for mitotic spindle pole formation and disintegration. When cells enter mitosis, PCNT is phosphorylated by PLK1 and recruits other PCM proteins to assemble spindle poles with a high microtubule organizing activity^10^. At the end of mitosis, PCNT is specifically cleaved by separase and removed from the centrosome^21^,^22^. This event is considered to be important for centriole separation during mitotic exit.

PLK1 is a mitotic kinase that participates in diverse mitotic events, such as sister chromatid separation, spindle assembly checkpoint and cytokinesis^23^,^24^. PLK1 is also localized at the centrosome and involved in multiple centrosomal functions^25^,^26^,^27^,^28^. However, it is largely unknown how PLK1 regulates centriole functions and what are critical substrates of PLK1 for centriole regulation. We previously reported that PLK1 phosphorylates PCNT at S1235 and S1241 residues for initiation of centrosome maturation^10^. In this report, we reveal that PLK1 phosphorylation is also essential for the separase-dependent cleavage of PCNT during mitotic exit. Our results propose molecular mechanisms how PLK1 controls centriole separation and eventually how centriole biogenesis is controlled during the cell cycle.

## Results

### BI2536 blocks both PCM disassembly and centriole separation

**Figure 1:**
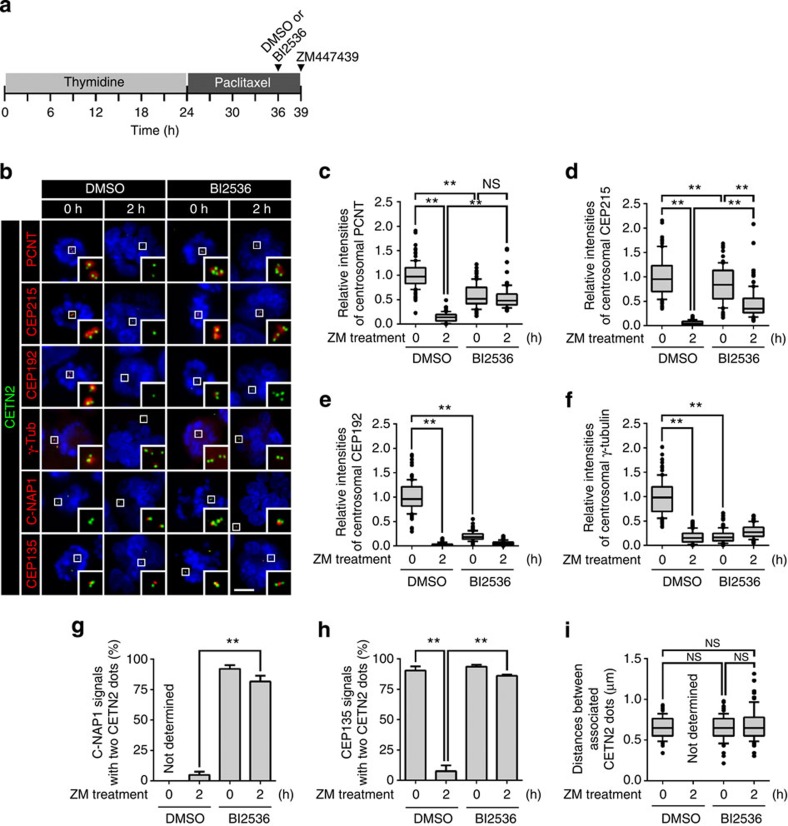
PLK1 regulation of PCM disassembly and centriole separation during mitotic exit. (**a**) HeLa cells were arrested at M phase with sequential treatment of thymidine and paclitaxel. BI2536 was added for the last 3 h and then ZM447439 (ZM) for 2 h to induce mitotic exit. (**b**) The cells were fixed before and after the ZM447439 treatment and co-immunostained with the centrin-2 antibody (CETN2, green), along with the PCNT, CEP215, CEP192, γ-tubulin (γ-Tub), C-NAP1 and CEP135 antibodies (red). DNA was visualized with 4,6-diamidino-2-phenylindole (blue). Scale bar, 10 μm. (**c**–**f**) Centrosomal intensities of the PCNT (**c**), CEP215 (**d**), CEP192 (**e**) and γ-tubulin (**f**) signals were shown with the box-and-whisker plot. *n*=90 per group in three independent experiments. (**g**,**h**) Centriole association was determined with 2:1 ratio of the centrin-2 and C-NAP1 (**g**) or CEP135 (**h**) dots. The C-NAP1 signals at prometaphase were undetectable in the absence of BI2536 (ref. 45). *n*=300 per group in three independent experiments. Values are means with s.d.'s. (**i**) Distance between associated centrioles in Fig. 1h was measured using the centrin-2 signals. Centriole was hardly associated in dimethylsulphoxide (DMSO)-treated G1 phase cells. *n*=90 per group in three independent experiments. The statistical significance was determined by unpaired two-tailed *t*-test in Prism 6 (not significant (NS), *P*>0.05; *P<0.05; **P<0.01).

To determine involvement of PLK1 in PCM disassembly during mitotic exit, we treated BI2536, a PLK1 inhibitor, to the M-phase-arrested HeLa cells. The cells were forced to exit mitosis with ZM447439, an aurora kinase inhibitor^29^, and immunostained with antibodies against selected PCM proteins (Fig. 1a). The BI2536 treatment reduced centrosomal CEP192 and γ-tubulin to the basal levels even before mitotic exit (Fig. 1b,e,f). However, the centrosomal levels of PCNT and CEP215 remained relatively abundant in the BI2536-treated cells even after mitotic exit (Fig. 1b–d). These results imply that the PLK1 activity is required for removal of PCNT and CEP215 from the centrosome during mitotic exit.

Next, we determined effects of BI2536 on centriole separation during the mitotic exit. Centriole association was determined with 2:1 ratio of centrin-2 and the proximal centriole markers, such as C-NAP1 and CEP135 (refs 3, 21; Fig. 1b). As expected, most of the daughter centrioles were separated from the mother centrioles once the cells were forced to exit mitosis (Fig. 1b,g,h). However, BI2536 blocked centriole separation in this condition (Fig. 1b,g,h). The distance between the associated centrioles was determined with the centrin-2 dots. Average distance between associated centrioles was <1 μm, and it did not significantly change after mitosis when BI2536 was treated (Fig. 1i). These results are consistent with the previous report that the PLK1 activity is required for centriole separation during mitotic exit^28^.

### PLK1 phosphorylation is necessary for PCNT cleavage

**Figure 2:**
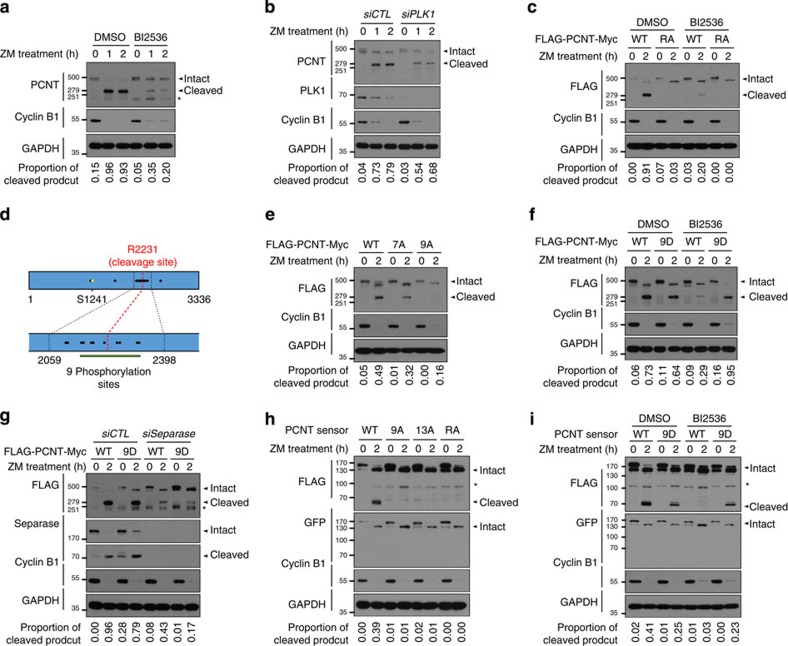
PLK1 regulation of the separase-dependent cleavage of PCNT during mitotic exit. (**a**) HeLa cells were treated with BI2536 during mitotic exit and forced to mitotic exit with ZM447439 (ZM). Lysates were subjected to immunoblot analyses with antibodies specific to PCNT, cyclin B1 and GAPDH. (**b**) Immunoblot analysis was carried out to determine the PCNT cleavage in PLK1-depleted HeLa cells during the forced mitotic exit. (**c**) Specific cleavage of ectopic FLAG-PCNT-Myc (WT) and FLAG-PCNT^R2231A^-Myc (RA) was determined in the presence of BI2536. (**d**) Summary of the PLK1 phosphorylation of PCNT. PLK1 phosphorylates 22 specific sites of PCNT (Supplementary Fig. 1) and these sites are marked with circles. Among them, nine phosphorylation sites (T2154, T2160, S2183, S2189, S2222, S2259, S2267, S2318 and T2324; green line) near the separase cleavage site (R2231; red) are critical for the PCNT cleavage. Two sites at the N-terminal end (S1235 and S1241; yellow circles) are essential for centrosome maturation. (**e**) Immunoblot analyses were carried out to determine specific cleavage of ectopic FLAG-PCNT^7A^-Myc (7A) and FLAG-PCNT^9A^-Myc (9A) during the forced mitotic exit. (**f**,**g**) Specific cleavage of ectopic FLAG-PCNT-Myc (WT) and FLAG-PCNT^9D^-Myc (9D) was determined in the presence of BI2536 (**f**) and in the separase-depleted cells (**g**). (**h**,**i**) Immunoblot analysis was carried out to determine the specific cleavage of the FLAG-mCherry-PCNT^2059–2398^-GFP-PACT fusion proteins with indicated phospho- (9A and 13A) and cleavage-resistant (RA) point mutations (**h**), and with phospho-mimetic mutation (9D) in the presence of BI2536 (**i**). The experiments were independently repeated at least twice. Asterisk, a non-specific band.

It is known that PCNT is specifically cleaved by separase and removed from the centrosome during mitotic exit^21^,^22^. To test whether PLK1 controls the separase-dependent cleavage of PCNT, we performed immunoblot analyses of PCNT in the BI2536-treated cells. The results showed that the specific cleavage of PCNT was suppressed in the BI2536-treated cells during mitotic exit (Fig. 2a). The specific PCNT cleavage was also suppressed in the PLK1-depleted cells (Fig. 2b). Finally, the cleavage of ectopic PCNT protein (FLAG-PCNT-Myc) was significantly reduced in the presence of BI2536, while the cleavage-resistant FLAG-PCNT^R2231A^-Myc mutant remained intact at all time (Fig. 2c). These results indicate that the PLK1 activity is necessary for the separase-dependent cleavage of PCNT during mitotic exit.

We identified 22 specific phosphorylation sites of PLK1 within PCNT after a series of *in vitro* kinase assays^10^ (Fig. 2d; Supplementary Fig. 1). Two of them at the N-terminal end (S1235 and S1241) have been known to be essential for centrosome maturation^10^ (Fig. 2d). To determine which phosphorylation sites are responsible for the separase-dependent cleavage of PCNT during mitotic exit, we performed a series of PCNT cleavage assays with HeLa cells that expressed the phospho-resistant mutants of FLAG-PCNT-Myc in both transient and stable manners. All the phospho-resistant FLAG-PCNT-Myc proteins were localized at the centrosome (Supplementary Fig. 2a). The results showed that the nine phosphorylation sites near the cleavage site (R2231) are responsible for the separase-dependent cleavage of PCNT during mitotic exit (Fig. 2e; Supplementary Fig. 3a–c). All examined combinations of phospho-resistant mutants within the nine sites were not as effective as FLAG-PCNT^9A^-Myc was, although FLAG-PCNT^S2259A^-Myc and FLAG-PCNT^S2259/2267A^-Myc showed some resistance to the specific cleavage (Supplementary Fig. 3d–g). These results suggest that multiple phosphorylation of PCNT near the cleavage site is necessary for the separase-dependent cleavage during mitotic exit. To confirm that PCNT phosphorylation at the nine residues is sufficient for separase-dependent cleavage of PCNT, we generated a HeLa cell line stably expressing FLAG-PCNT^9D^-Myc in which the nine phosphorylation sites were substituted to aspartic acid (Supplementary Fig. 2b). The results showed that the phospho-mimetic PCNT mutant was efficiently cleaved during mitotic exit even in the presence of BI2536 (Fig. 2f). However, the specific cleavage of the phospho-mimetic PCNT mutant was significantly reduced in separase-depleted cells (Fig. 2g). These results collectively propose that the PLK1 phosphorylation at multiple sites of PCNT is essential for the separase-dependent cleavage of PCNT during mitotic exit.

Agircan and Schiebel^30^ recently developed the PCNT sensor protein for detection of separase-dependent cleavage of PCNT at the centrosome. The construct includes 2059–2398 residues of PCNT which contains 13 phosphorylation sites (Fig. 4d; Supplementary Fig. 4a). We examined the importance of PLK1 phosphorylation on PCNT cleavage using the PCNT sensor protein (FLAG-mCherry-PCNT^2059–2398^-GFP-PACT). All PCNT sensor proteins were localized at the centrosome (Supplementary Fig. 4b). As expected, the wild-type PCNT sensor protein was efficiently cleaved during mitotic exit, while cleavage-resistant PCNT sensor protein was not (Fig. 2h). In this condition, we observed a significant reduction of the specific cleavage of phospho-resistant PCNT sensor proteins (Fig. 2h). Furthermore, the phospho-mimetic PCNT sensor protein was cleaved even in the presence of BI2536 (Fig. 2i). The C-terminal fragments of the PCNT sensor proteins were not detected with the green fluorescent protein (GFP) antibody, implying that it is rapidly degraded by the N-end rule pathway (Fig. 2h,i). These results strongly support our proposal that multiple phosphorylation of PCNT near the cleavage site is a necessary step for separase-dependent cleavage of PCNT during mitotic exit.

### PLK1 phosphorylates PCNT *in vivo*

**Figure 3:**
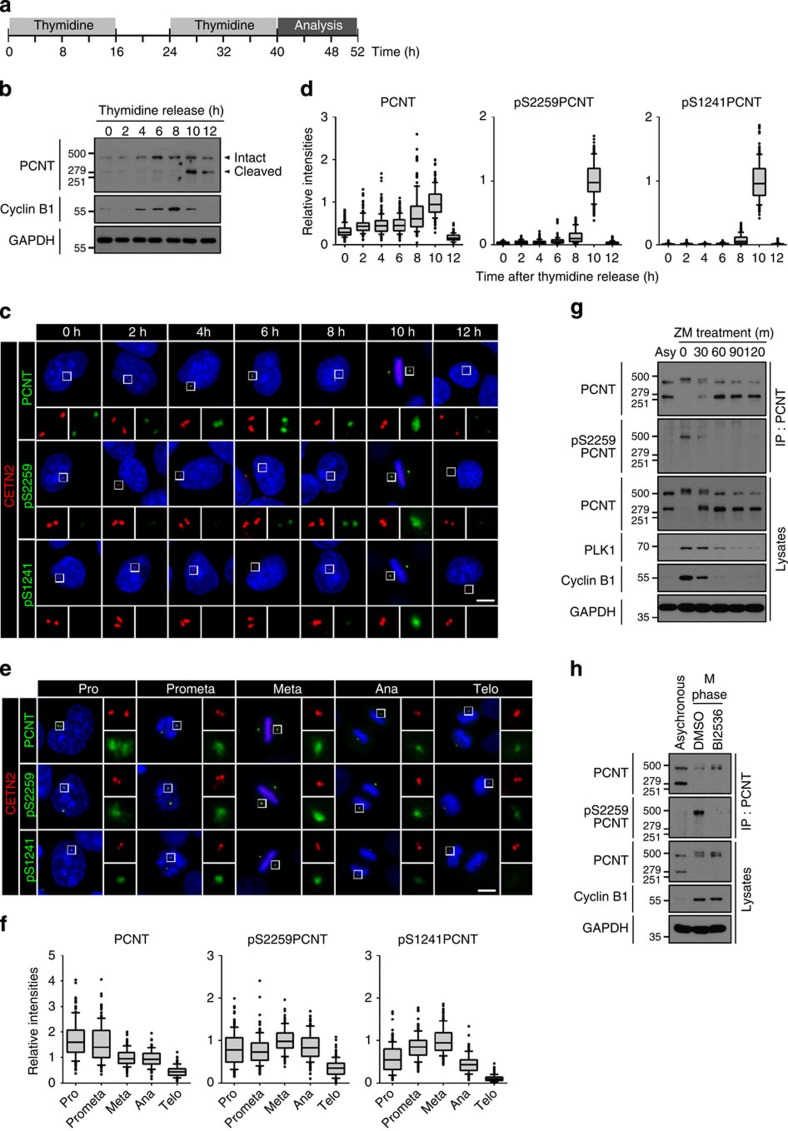
PLK1 phosphorylation of PCNT *in vivo*. (**a**) HeLa cells were synchronously released from G1/S phase with the double thymidine block method and collected every 2 h for the immunoblot and immunostaining analyses. (**b**) The cells were subjected to immunoblot analyses with the PCNT, cyclin B1 and GAPDH antibodies. (**c**) Co-immunostaining analysis was performed with the centrin-2 antibody (CETN2, red) along with the PCNT and phospho-PCNT (pS2259PCNT and pS1241PCNT) antibodies (green). Scale bar, 10 μm. (**d**) Centrosomal intensities of PCNT and phospho-PCNT were densitometrically determined and shown with the box-and-whisker plot. *n*=153 per group in three independent experiments. (**e**) Co-immunostaining analysis of mitotic HeLa cells was performed with indicated antibodies. The mitotic stages were determined with 4,6-diamidino-2-phenylindole-staining patterns. Scale bar, 10 μm. (**f**) Centrosomal intensities of PCNT and phospho-PCNT were determined in cells at specific mitotic stages. *n*=153 per group in three independent experiments. (**g**) M-phase-arrested HeLa cells were forced to exit mitosis with ZM447439 (ZM). At indicated time points, the cells were subjected to immunoprecipitation (IP) followed by immunoblot analyses with antibodies specific to PCNT, pS2259PCNT, PLK1, cyclin B1 and GAPDH. (**h**) The M-phase-arrested HeLa cells were treated with BI2536 for 3 h and subjected to IP followed by immunoblot analyses with indicated antibodies.

To determine specific phosphorylation of PCNT *in vivo*, we generated a phospho-antibody specific to pS2259 of PCNT (pS2259PCNT; Supplementary Fig. 5a). Immunoblot analyses with PCNT immunoprecipitants showed that pS2259PCNT-specific band was detected only in mitotic cells (Supplementary Fig. 5b). Furthermore, pS2259PCNT-specific signals at the centrosome were only restored in cells rescued with FLAG-PCNT-Myc, but not with phospho-resistant FLAG-PCNT^S2259A^-Myc (Supplementary Fig. 5c,d). To more precisely observe specific phosphorylation of PCNT according to cell cycle, we synchronously released HeLa cells from double thymidine block and compared it with another phospho-PCNT antibody against pS1241 of PCNT (pS1241PCNT), which was previously characterized for centrosome maturation^10^ (Fig. 3a). Immunoblot analysis revealed that the specific cleavage bands of PCNT was detected at the 10th hour with cyclin B1 degradation indicating that they reached the M phase at the 10th hour and exited mitosis at the 12th hour (Fig. 3b). Immunostaining analyses with the identical set of synchronous HeLa cells revealed that the centrosomal PCNT levels increased at the 10th hour and decreased afterwards (Fig. 3c,d). The centrosomal levels of both pS1241PCNT and pS2259PCNT also peaked at the 10th hour but they were more marked than the PCNT protein (Fig. 3c,d). These results suggest that the centrosomal PCNT is simultaneously phosphorylated at both S1241 and S2259 during M phase.

We determined the phospho-PCNT levels at specific stages of mitosis. The immunostaining analyses revealed that the centrosomal signals of phospho-PCNT, as well as PCNT were the highest at early mitosis and decreased at telophase (Fig. 3e,f). Immunoblot analyses with PCNT immunoprecipitants showed that the pS2259PCNT-specific band was only detected at early time points after the ZM447439 treatment (Fig. 3g). Furthermore, BI2536 significantly reduced the intensity of the pS2259PCNT-specific band in M-phase-arrested cells (Fig. 3h). These results indicate that PLK1 phosphorylation precedes the PCNT cleavage during mitotic exit.

### PCNT phosphorylation is necessary for centriole separation

**Figure 4:**
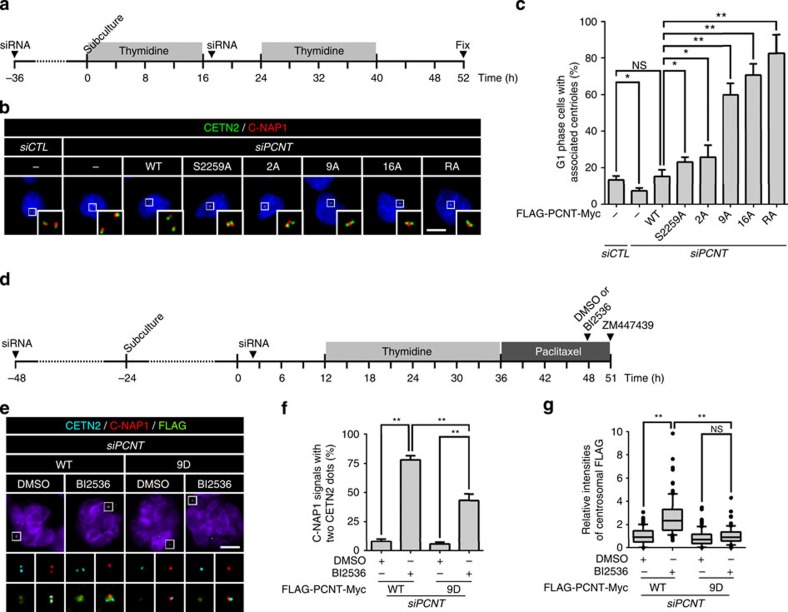
PLK1 phosphorylation of PCNT for centriole separation during mitotic exit. (**a**) Endogenous PCNT was depleted in the stable cell lines expressing FLAG-PCNT-Myc proteins. As a result, endogenous PCNT was rescued with the ectopic PCNT proteins (Supplementary Fig. 5a,b). The cell cycle was synchronized with the double thymidine block and release. (**b**) The PCNT-rescued cells were subjected to co-immunostaining analyses with antibodies specific to C-NAP1 (red) and centrin-2 (CETN2, green). Scale bar, 10 μm. (**c**) Centriole association in G1 phase cells was determined with the 2:1 ratio of centrin-2 and C-NAP1. Cells with only two centrin-2 dots were determined in G1 phase. Values are means with s.d.'s. *n*=300 per group in three independent experiments. (**d**) The cells rescued with FLAG-PCNT-Myc (WT) or FLAG-PCNT^9D^-Myc (9D) were arrested at M phase, treated with BI2536 to block the PLK1 activity, and with ZM447439 (ZM) to force mitotic exit. (**e**) The HeLa cells rescued with FLAG-PCNT^9D^-Myc (9D) were subjected to co-immunostaining analyses with antibodies specific to centrin-2 (cyan), C-NAP1 (red) and FLAG (green). Scale bar, 10 μm. (**f**) Centriole association was determined with the 2:1 ratio of centrin-2 and C-NAP1. Values are means with s.d.'s. *n*=300 per group in three independent experiments. (**g**) The centrosomal levels of ectopic FLAG-PCNT-Myc were statistically analysed and shown with the box-and-whisker plot. *n*=90 per group in three independent experiments. The statistical significance was determined by unpaired two-tailed *t*-test in Prism 6 (not significant (NS), *P*>0.05; *P<0.05; **P<0.01).

To examine whether PLK1 phosphorylation of PCNT is necessary for centriole separation during mitotic exit, we prepared PCNT-rescued cells (Supplementary Fig. 6a,b). In this study, we carried out all knockdown-rescue experiments by depleting endogenous PCNT in cells that stably expressed short interfering RNA (siRNA)-resistant FLAG-PCNT-Myc proteins (Supplementary Fig. 6). To avoid unexpected effects of prolonged mitotic arrest and ZM447439, we synchronized the cell cycle at early G1 phase using double thymidine block release method (Fig. 4a). G1 phase cells were determined with two centrin-2 dots and centriole association was determined with 2:1 ratio of centrin-2 and C-NAP1. As expected, most centrioles in the PCNT-depleted cells rescued with wild-type FLAG-PCNT-Myc were separated, while those with cleavage-resistant FLAG-PCNT^R2231A^-Myc remained associated after mitosis (Fig. 4b,c). In this condition, the proportion of cells with the associated centrioles increased, depending on the number of alanine substitutes within the ectopic PCNT of the rescued cells (Fig. 4b,c). We also observed that phospho-mimetic FLAG-PCNT^9D^-Myc partially rescued centriole separation in BI2536-treated cells (Fig. 4d–f). These results imply that specific phosphorylation of PCNT is prerequisite for centriole separation during mitotic exit. The sum of specific phosphorylation near the cleavage site proportionally affects PCNT cleavage and eventually leads to centriole separation.

### PCNT and CEP215 are essential for centriole association

**Figure 5:**
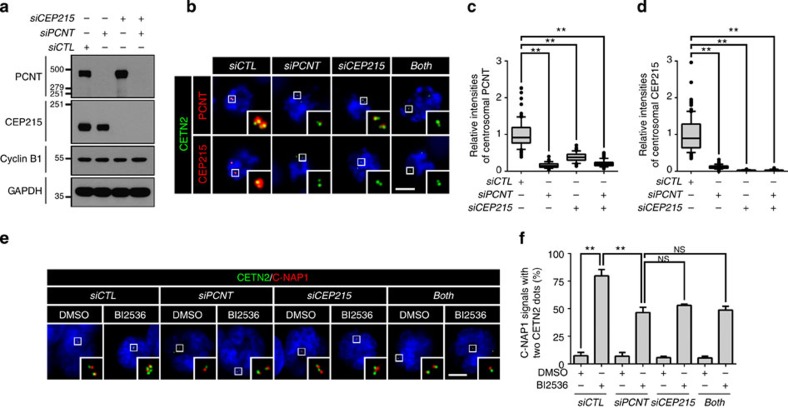
Centriole separation in the PCNT- and/or CEP215-depleted cells. (**a**–**d**) Depletion of PCNT and CEP215 was confirmed with immunoblot (**a**) and co-immunostaining (**b**) with the PCNT and CEP215 antibodies. Scale bar, 10 μm. (**c**,**d**) Centrosomal intensities of PCNT and CEP215 in mitotic cells were determined and shown with the box-and-whisker plot. *n*=90 per group in three independent experiments. (**e**) The PCNT- and/or CEP215-depleted cells were arrested at M phase, treated with BI2536 for 3 h, forced to exit mitosis with ZM447439 (ZM) for 2 h and co-immunostained with antibodies specific to centrin-2 (CETN2, green) and C-NAP1 (red). Scale bar, 10 μm. (**f**) Centriole association was determined with the 2:1 ratio of centrin-2 and C-NAP1. Values are means with s.d.'s. *n*=300 per group in three independent experiments. The statistical significance was determined by unpaired two-tailed *t*-test in Prism 6 (not significant (NS), *P*>0.05; *P<0.05; **P<0.01).

PCNT and CEP215 are considered key components of the PCM^31^. It was recently reported that depletion of CEP215 from the centrosome is critical for centriole separation during mitotic exit^32^. To determine whether the PLK1 activity is still critical for separation of centrioles with limited PCM components, we treated BI2536 in the PCNT- and/or CEP215-depleted cells. Depletion of either PCNT or CEP215 did not affect one another in total cellular levels at M phase (Fig. 5a). However, the centrosomal levels of both proteins were significantly reduced in mitotic cells of any depletion of the proteins (Fig. 5b–d). This result reflects that CEP215 and PCNT are interdependent to localize at the mitotic spindle poles^31^. We determined centriole separation of PCNT- and/or CEP215-depleted cells in the presence of BI2536. The results showed that centrioles of the PCNT- and/or CEP215-depleted cells, as well as control cells were separated during mitotic exit (Fig. 5e,f). BI2536 significantly inhibited centriole separation in the control cells, but not so much in the PCNT- and/or CEP215-depleted cells (Fig. 5e,f). No significant difference in the proportion of centriole separation was observed among the PCNT- and/or CEP215-depleted cells (Fig. 5e,f). These results support the notion that PLK1 phosphorylation of PCNT and eventual disintegration of PCM are essential for centriole separation. Furthermore, the results imply that another substrate of PLK1 is involved in centriole disengagement and separation.

**Figure 6:**
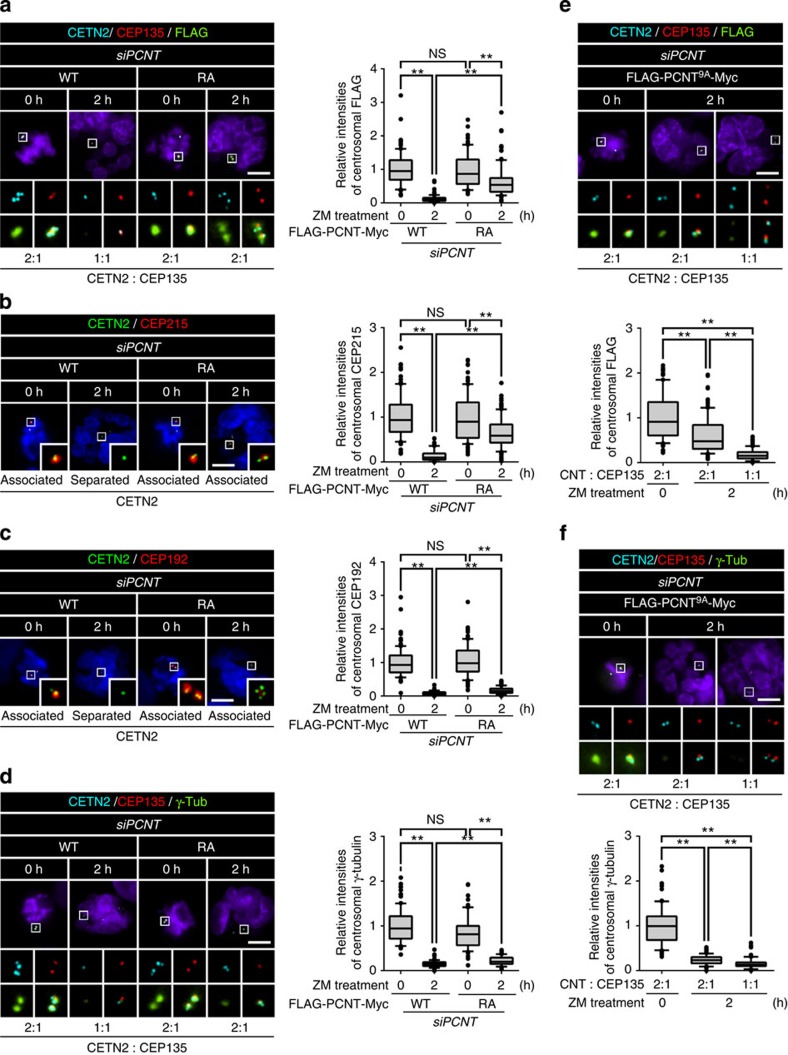
Importance of PCM for maintaining centriole association. (**a**–**f**) The M-phase-arrested cells rescued with FLAG-PCNT-Myc (WT) or FLAG-PCNT^R2231A^-Myc (RA) (**a**–**d**) and FLAG-PCNT9 A-Myc (9A) (**e**,**f**) were forced to exit mitosis with ZM447439 (ZM) and co-immunostained with indicated antibodies. Scale bars, 10 μm. Centrosomal intensities of the indicated proteins were determined before and after the ZM447439 treatment and shown with the box-and-whisker plot. Centriole association was determined with the 2:1 ratio of centrin-2 and CEP135 dots in **a** and **d**–**f**. The centriole separation in **b** and **c** was determined, based on the proximity of centriole pairs (<1 μm). *n*=90 per group in 3 independent experiments. The statistical significance was determined by unpaired two-tailed *t*-test in Prism 6 (not significant (NS), *P*>0.05; *P<0.05; **P<0.01).

We tested the hypothesis in which the PCNT cleavage leads to disassembly of PCM components followed by centriole separation during mitotic exit. The hypothesis predicts that both PCNT and CEP215 should remain at the centrosome if PCNT were not cleaved during mitotic exit. To test the hypothesis, we determined the centrosomal levels of selected PCM proteins in cells rescued with the cleavage-resistant FLAG-PCNT^R2231A^-Myc. As expected, all the tested PCM proteins in the FLAG-PCNT-Myc-rescued cells were reduced to basal level after mitotic exit (Fig. 6a–d). The centrosomal levels of CEP192 and γ-tubulin were also reduced in the FLAG-PCNT^R2231A^-Myc-rescued cells during mitotic exit even with the centrioles associated (Fig. 6c,d). However, the centrosomal levels of PCNT and CEP215 remained relatively high in the FLAG-PCNT^R2231A^-Myc-rescued cells with centriole associated even after mitosis (Fig. 6a,b). These results suggest that centrioles are associated as far as both PCNT and CEP215 remain at PCM even after mitotic exit.

Next, we determined the centrosomal levels of the PCM proteins with associated or separated centrioles in the cells rescued with phospho-resistant FLAG-PCNT^9A^-Myc (Fig. 6e,f). As expected, majority of the rescued cells had centrioles associated and only a small fraction of the cells had centrioles separated at the end of mitosis (Fig. 6e,f). The centrosomal levels of the ectopic PCNT proteins were higher in the centrosomes with associated centrioles than those with separated centrioles (Fig. 6e). The centrosomal levels of γ-tubulin, however, were reduced to basal levels, irrespective of the centriole status (Fig. 6f). These results support the hypothesis that the centrosomal PCNT and CEP215 are essential for maintaining centriole association during mitosis. In consistent with this notion, we observed that PCNT was removed from the FLAG-PCNT^9D^-Myc-rescued cells even in the presence of the PLK1 inhibitor (Fig. 4f).

### Dual functions of PLK1 phosphorylation of PCNT

**Figure 7:**
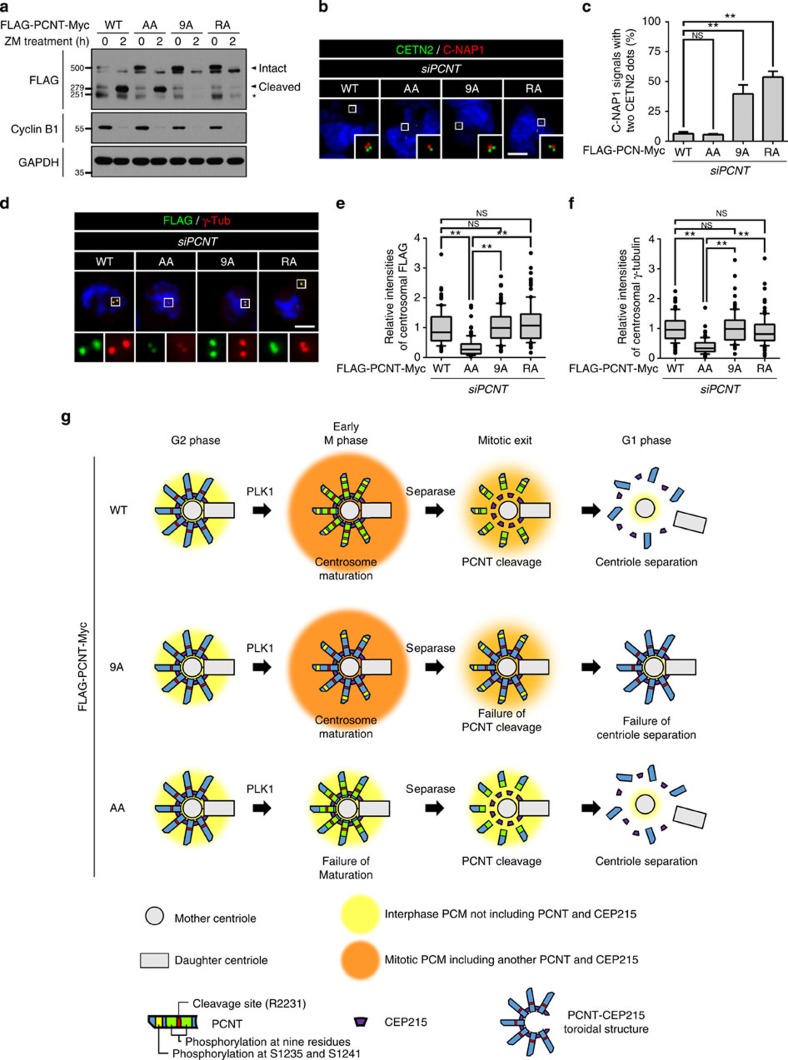
PLK1 control for dual functions of PCNT. (**a**) HeLa cells were transiently transfected with the wild-type and mutant FLAG-PCNT-Myc (WT, AA, 9A and RA), arrested at M phase and forced to exit mitosis with ZM447439 (ZM). Immunoblot analyses were performed to determine specific cleavage of FLAG-PCNT-Myc during mitotic exit. Asterisk, a non-specific band. (**b**) The PCNT-depleted cells were rescued with the wild-type and mutant FLAG-PCNT-Myc, and subjected to co-immunostaining analyses with antibodies specific to centrin-2 (CETN2, green) and C-NAP1 (red). Scale bar, 10 μm. (**c**) Centriole association was determined with the 2:1 ratio of centrin-2 and C-NAP1 dots. Values are means with s.d.'s. *n*=300 per group in three independent experiments. (**d**) The PCNT-rescued cells were co-immunostained with antibodies against FLAG (green) and γ-tubulin (red). Scale bar, 10 μm. (**e**,**f**) Centrosomal intensities of ectopic FLAG-PCNT-Myc (**e**) and γ-tubulin (**f**) were determined. *n*=90 per group in three independent experiments. The statistical significance was determined by unpaired two-tailed *t*-test in Prism 6 (not significant (NS), *P*>0.05; *P<0.05; **P<0.01). (**g**) Model. PLK1 phosphorylation of PCNT induces centrosome maturation in early M phase and PCNT cleavage during mitotic exit. As a result, centrioles are separated from each other with little PCM surrounded. The centrosomes in PCNT^9A^-rescued cells were properly matured in early M phase but PCNT was not cleaved by separase during mitotic exit. As a result, centrioles remained associated within the PCM scaffold. The centrosomes in PCNT^AA^-rescued cells were not matured in early M phase but PCNT was cleaved by separase during mitotic exit. As a result, centrioles are separated from each other with little PCM surrounded.

We previously reported that PLK1 phosphorylation of PCNT at S1235 and S1241 residues is an initial step for centrosome maturation at the onset of mitosis^10^. In this work, we revealed that PLK1 phosphorylation near the cleavage site of PCNT is critical for the PCNT cleavage. We examined whether these two events are independent or not. First, we observed that FLAG-PCNT^AA^-Myc in which the serine residues for centrosome maturation (S1235 and S1241) were substituted to alanine was specifically cleaved during mitotic exit (Fig. 7a). Furthermore, we carried out knockdown-rescue experiments with stable cell lines expressing FLAG-PCNT^AA^-Myc (Supplementary Figs 2c and 6e). The results showed that the centrioles were properly separated after mitosis when endogenous PCNT was rescued with FLAG-PCNT^AA^-Myc (Fig. 7b,c). Second, we observed that both FLAG-PCNT^9A^-Myc and FLAG-PCNT^R2231A^-Myc were properly placed to the centrosome during mitotic entry (Fig. 7d,e). Furthermore, the centrosomal γ-tubulin levels were effectively rescued with both FLAG-PCNT^9A^-Myc and FLAG-PCNT^R2231A^-Myc, but not with FLAG-PCNT^AA^-Myc (Fig. 7d,f). These results indicate that PLK1 phosphorylation of PCNT independently governs two mitotic events: one is centrosome maturation at the onset of mitosis, and the other is the PCNT cleavage during mitotic exit.

## Discussion

In this report, we revealed that PCNT should be phosphorylated by PLK1 to be a suitable substrate of separase. It is known that the separase-dependent cleavage of PCNT is an inevitable event for centriole separation during mitotic exit^21^,^22^. Therefore, we propose that PLK1 phosphorylation is a priming step for PCNT cleavage and eventually for centriole separation during mitotic exit (Fig. 7g). Our results are consistent with the previous reports in which the PLK1 activity is required for centriole separation during mitotic exit^28^.

It is well known that separase is activated at the anaphase onset when securin and cyclin B1 are degraded by the anaphase-promoting complex (APC/C)^33^,^34^. However, a residual amount of the separase activity is also detected before the anaphase and even in interphase cells^30^,^35^. The PLK1-dependent phosphorylation would preclude premature centriole separation, which might result in multipolar spindle formation and mitotic catastrophe. Therefore, PLK1 phosphorylation of PCNT provides an additional layer of regulatory mechanism to ensure the timeliness and fidelity of centriole separation during mitotic exit. It was recently reported that PP2A forms a complex with inactive separase^36^. It is possible that PCNT may be a substrate of the separase-bound PP2A. If this is the case, centriole separation mechanisms would be additionally fine-tuned with a counterbalance between PLK1 and PP2A for the separase-dependent cleavage of PCNT. Our results are consistent with the proposal that PLK1 and APC/C activities cooperatively function for centriole separation in the short time-window of the cells finishing mitosis^37^.

PCNT and CEP215 are major PCM components in the mitotic spindle poles as well as in the interphase centrosomes^31^. Once PCNT is cleaved by separase, the C-terminal fragment of PCNT is immediately degraded following the N-end rule pathway^21^. Since both the CEP215-interacting and the centrosome-targeting domains reside in the C-terminal fragment of PCNT, the separase-dependent cleavage of PCNT results in disassembly of itself from centrosome and subsequently centriole separation during mitotic exit (Fig. 7g). Depletions of either one or both of PCNT and CEP215 probably limit the assembly of PCM scaffold that plays critical roles in maintaining centriole association (Fig. 5b–d). Consistent with this notion, CEP215 depletion results in centriole separation in the cells even with a non-cleavable PCNT mutant^32^.

Two different models for the mother and daughter centriole association have been proposed. First, the orthogonal configuration of engaged centrioles is maintained by a glue protein that may form a linker between the mother and daughter centriole^38^. Second, the mother and daughter centrioles are trapped within PCM so that centrioles remain associated^4^,^21^,^22^,^32^. These two models are not mutually exclusive, so that it is possible that daughter centriole is disengaged but trapped within the PCM until the PCM scaffold is disassembled^32^,^39^. In this study, we revealed that PLK1 is critical for centriole separation by regulating PCM disassembly during mitotic exit. However, PLK1 may be also involved in the centriole disengagement to break orthogonal arrangement before the centriole separation. In fact, PLK1 depletion prevented centriole disengagement and reduplication in S-phase-arrested U2OS cells^26^,^37^. Centriole disengagement and reduplication in G2-arrested cells also depend on PLK1 activity^27^. Finally, when PLK1 activity is inhibited in late G2 phase, but not in late M phase, centriole disengagement is completely blocked in the separase-null cells^28^. These results suggest that PLK1 regulates both centriole disengagement and centriole separation events. We identified that PCNT is a key substrate of PLK1 for centriole separation. However, it remains to be identified what is a PLK1 substrate for centriole disengagement.

It is known that PLK1 phosphorylation of PCNT at S1231 and S1241 residues are required for centrosome maturation^10^. Here we report that another set of PCNT residues should be phosphorylated by PLK1 for centriole separation. Therefore, two different functions of PCNT are induced by PLK1 phosphorylation at distinct sites of PCNT (Fig. 7g). It is well known that PLK1 carries out multiple mitotic functions by phosphorylating diverse substrates. Our results provide an example that PLK1 can carry out multiple functions by phosphorylating specific sets of residues within a single protein species.

## Methods

### Plasmids, siRNA and cell culture

All PCNT (NCBI reference sequence: NM_006031.5) constructs were subcloned into pLVX-IRES-Puro vector (Clontech, 632183) and tagged with 3 × FLAG and Myc at 5′- and 3′-ends, respectively. To generate siRNA-resistant PCNT construct, we induced silent mutations of PCNT with the following primers: 5′-GCA GCA GAA CTC AAG GAG-3′ and 5′-TGG ACC TCT TCG AAT GAG-3′.

To make PCNT sensor (3 × FLAG-mCherry-PCNT^2059–2398^-GFP-PACT), we linked the PCNT^2059–2398^ fragment with mCherry and GFP at 5′- and 3′-ends, respectively. The PACT domain of PCNT^3112–3336^ was joined at 3′-end of GFP. In addition, the 3 × FLAG fragment was linked at 5′-end of mCherry.

For depletion of endogenous PLK1 and PCNT, *siPLK1* (5′-AAG CGG GAC TTC CTC ACA TCA-3′; ref. 40) and *siPCNT* (5′-UGG ACG UCA UCC AAU GAG A-3′; ref. 14) were used. A scrambled siRNA sequence (*siCTL*; 5′-GCA AUC GAA GCU CGG CUA CTT-3′) was used as a control. RNAiMAX (Invitrogen, 13778-075) was used for siRNA transfection according to the manufacturer's protocol.

The HeLa cells were cultured in DMEM (Welgene, LM 001–05) supplemented with 10% fetal bovine serum (Welgene, S101-01) and antibiotics (Invivogen, ANT-MPT) at 37 °C and 5% CO_2_. To generate stable cell lines, we seeded 2.4 × 10^5^ HeLa cells on a 60-mm dish and 2.5 μg of plasmid DNA was transfected in the next day. The plasmids were transfected into the HeLa cells using Fugene HD (Promega, E2311). One day after the transfection, the cells were transferred to a 100-mm dish and treated with 1 mg ml^−1^ puromycin (Calbiochem, 540222) for 2–3 weeks. Monoclonal cell lines were established with the dilution cloning method and maintained with 0.5 mg ml^−1^ puromycin.

To synchronize cell cycle, we used 2 mM thymidine (Sigma, T9250), 5 μM paclitaxel (Sigma, T7402), and 2 μM ZM447439 (Cayman chemical company, 13601). For inhibition of PLK1 activity during mitosis, we treated the cells with 100 nM BI2536 (Selleck chemicals, S1109) for 3 h after the thymidine–paclitaxel block (Fig. 1a; Fig. 4d).

### Antibodies

The C-NAP1 (ref. 40; ICC 1:20,000), CEP135 (ref. 41; ICC 1:5,000), CEP215 (ref. 42; ICC 1:5,000, IB 1:5,000), PCNT^43^ (ICC 1:20000, IB 1:5,000) and pS1241PCNT^10^ (ICC 1:100) antibodies were previously described. Antibodies specific to centrin-2 (Merck Millipore, 04-1624; ICC 1:1,000), CEP192 (Bethyl Laboratories, A302-324A; ICC 1:20000), cyclin B1 (Santa Cruz Biotechnology, sc-254; IB 1:1,000), GFP (Santa Cruz Biotechnology, sc-9996; ICC 1:500, IB 1:500), GAPDH (Life Technologies, AM4300; IB 1:20,000), FLAG (Sigma-Aldrich, F3165; ICC 1:2000, IB 1:10,000), mCherry (Abcam, ab167453; ICC 1:1,000, IB 1:500), PLK1 (Zymed, 33-1700; IB 1:500), γ-tubulin (Santa Cruz Biotechnology, sc-7396; ICC 1:200) and separase (Abcam, ab16170; IB 1:500) were purchased. The secondary antibodies conjugated with fluorescent dye (Alexa-488, Alexa-594, and Alexa-647, Life Technologies; 1:1,000) and with horseradish peroxidase (Sigma-Aldrich or Millipore, 1:10,000) were purchased.

The pS2259PCNT antibodies (ICC 1:1,000, IB 1:100) were generated as described previously^44^. In brief, the KLH-conjugated ‘CSADT(pS)LGDRAD' peptide (synthesized by Abclon) was injected into rabbits with a complete Freud's adjuvant (Sigma-Aldrich, F5881). To induce strong immune response, the same peptide with an incomplete Freud's adjuvant (Sigma-Aldrich, F5506) was additionally injected twice. The immune serum was obtained and the phospho-antibody was affinity purified. All procedures with animals were approved by the Seoul National University Institutional Animal Care and Use Committees.

### Immunoprecipitation and immunoblot analyses

For immunoprecipitation, HeLa cells were lysed on ice for 20 min with a lysis buffer (150 mM NaCl, 0.1% Triton X-100, 20 mM Tris-HCl at pH 7.5, 10 mM NaF, 1 mM Na_3_VO_4_, 1 mM EDTA, 1 mM EGTA, 20 mM β-glycerophosphate, 5 mM MgCl_2_ and 5% glycerol) containing a protease inhibitor cocktail (GenDEPOT, P3100) and centrifuged at 12,000 r.p.m. for 20 min at 4 °C. The supernatants were incubated with antibody against PCNT for 4–6 h at 4 °C and then immunoprecipitated with Protein A bead (GE Healthcare Life Sciences, 17-0780-01) for 1 h at 4 °C. The PCNT immunoprecipitants were eluted from Protein A bead by adding the SDS sample buffer (62.5 mM Tris-HCl at pH 6.8, 2% SDS, 10% glycerol, 10 mM dithiothreitol (DTT) and 0.01% bromophenol blue) and boiled for 5 min. The samples were subjected to immunoblot analyses with indicated antibodies.

For immunoblot analyses, the cells were lysed on ice for 10 min with RIPA buffer (150 mM NaCl, 1% Triton X-100, 0.5% sodium deoxycolate, 0.1% SDS, 50 mM Tris-HCl at pH 8.0, 10 mM NaF, 1 mM Na_3_VO_4_, 1 mM EDTA and 1 mM EGTA) containing a protease inhibitor cocktail (Sigma-Aldrich, P8340) and centrifuged with 12,000 r.p.m. for 10 min at 4 °C. The supernatants were mixed with 4 × SDS sample buffer (250 mM Tris-HCl at pH 6.8, 8% SDS, 40% glycerol and 0.04% bromophenol blue) and 10 mM DTT (Amresco, 0281-25G). Mixtures were boiled for 5 min.

To detect intact and cleaved PCNT, 15–20 μg of proteins were loaded in SDS–polyacrylamide gel (3% stacking gel and 4% separating gel), electrophoresed and transferred to Protran BA85 nitrocellulose membranes (GE Healthcare Life Sciences, 10401196). The membranes were blocked with blocking solution (5% nonfat milk in 0.1% Tween 20 in TBS or 5% bovine serum albumin in 0.1% Tween 20 in TBS) for 2 h, incubated with primary antibodies diluted in blocking solution for 16 h at 4 °C, washed four times with TBST (0.1% Tween 20 in TBS), incubated with secondary antibodies in blocking solution for 30 min and washed again. To detect the signals of secondary antibodies, ECL reagent (ABfrontier, LF-QC0101) and X-ray films (Agfa, CP-BU NEW) were used. In the cases of other proteins, 5% stacking and 10% separating gels were used.

Uncropped film images of immunoblot are provided in Supplementary Fig. 7.

### Immunstaining analysis

For immunocytochemistry, cells seeded on cover glass were fixed with cold methanol for 10 min and washed three times with cold PBS. After incubation of PBST (0.1% Triton X-100 in PBS) for 10 min, the cells were blocked with blocking solution (3% bovine serum albumin, and 0.3% Triton X-100 in PBS) for 30 min, incubated with primary antibodies diluted in blocking solution for 1 h, washed three times with PBST, incubated with secondary antibodies in blocking solution for 30 min, washed twice with PBST, incubated with 4,6-diamidino-2-phenylindole solution for 3 min and washed twice with PBST. The cover glasses were mounted on a slide glass with ProLong Gold antifade reagent (Life Technologies, P36930). Images were acquired from fluorescence microscopies equipped with digital cameras (Olympus IX51 equipped with QImaging QICAM Fast 1394 or Olympus IX81 equipped with ANDOR iXon^EM^+) and processed in ImagePro 5.0 (Media Cybernetics) or MetaMorph 7.6 (Molecular Devices). Inset images were enlarged four times in Image J 1.49 (National Institutes of Health, USA) or Photoshop CS6 (Adobe) using option of bicubic interpolation. In the cases of quadruple staining, Image 5D (Fiji), which is a plug-in of Image J, was used in order for pseudo-colouring.

To measure fluorescence intensities, we immunostained all cells at the same time with same diluent antibodies. All images were captured at same exposure time without stopping. Image J 1.49 was used to measure fluorescence intensities at centrosomes. In each measurement, background signals were subtracted from the sum of fluorescence signals at centrosome.

### *In vitro* kinase assay

Methods for *in vitro* kinase assay have been previously described^10^. In brief, recombinant PLK1 and PCNT fragment proteins were mixed in a kinase assay buffer (50 mM Tris-HCl at pH 7.5, 10 mM MgCl_2_, 1 mM DTT and 5 μM ATP) with 0.25 μCi γ-[^32^P]ATP for 30 min at 30 °C.

### Statistical analysis

For statistical analysis, experiments were independently performed three times. To calculate *P* values, unpaired two-tailed *t*-test was performed in Prism 6 (GraphPad Software). All Measured fluorescent intensities were displayed with box-and-whisker plots in Prism 6 (lines, median; vertical boxes; values from 25th to 75th; down error bars, 10th value; up error bar, 90th value; circles, outliers).

## Additional information

**How to cite this article:** Kim, J. *et al.* PLK1 regulation of PCNT cleavage ensures fidelity of centriole separation during mitotic exit. *Nat. Commun.* 6:10076 doi: 10.1038/ncomms10076 (2015).

## Supplementary Material

Supplementary Information Supplementary Figures 1-7
