# Supplementary material for: PLK1 regulation of PCNT cleavage ensures fidelity of centriole separation during mitotic exit
Source: Nat Commun. 2015 Dec 9;6:10076. doi: 10.1038/ncomms10076 (PMC4682042; doi:10.1038/ncomms10076)

Supplementary Figure 1. PLK1 phosphorylation of PCNT *in vitro*

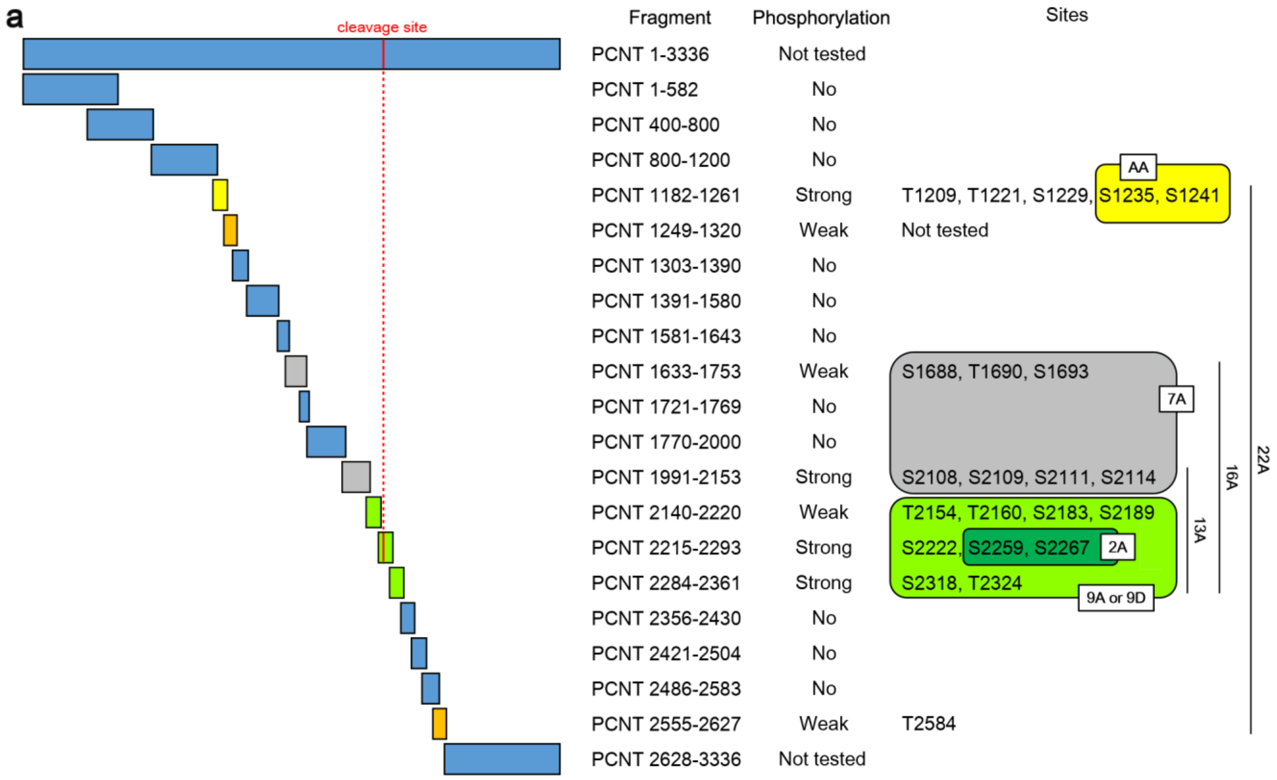

Supplementary Figure 1. PLK1 phosphorylation of PCNT *in vitro* (Cont'd)

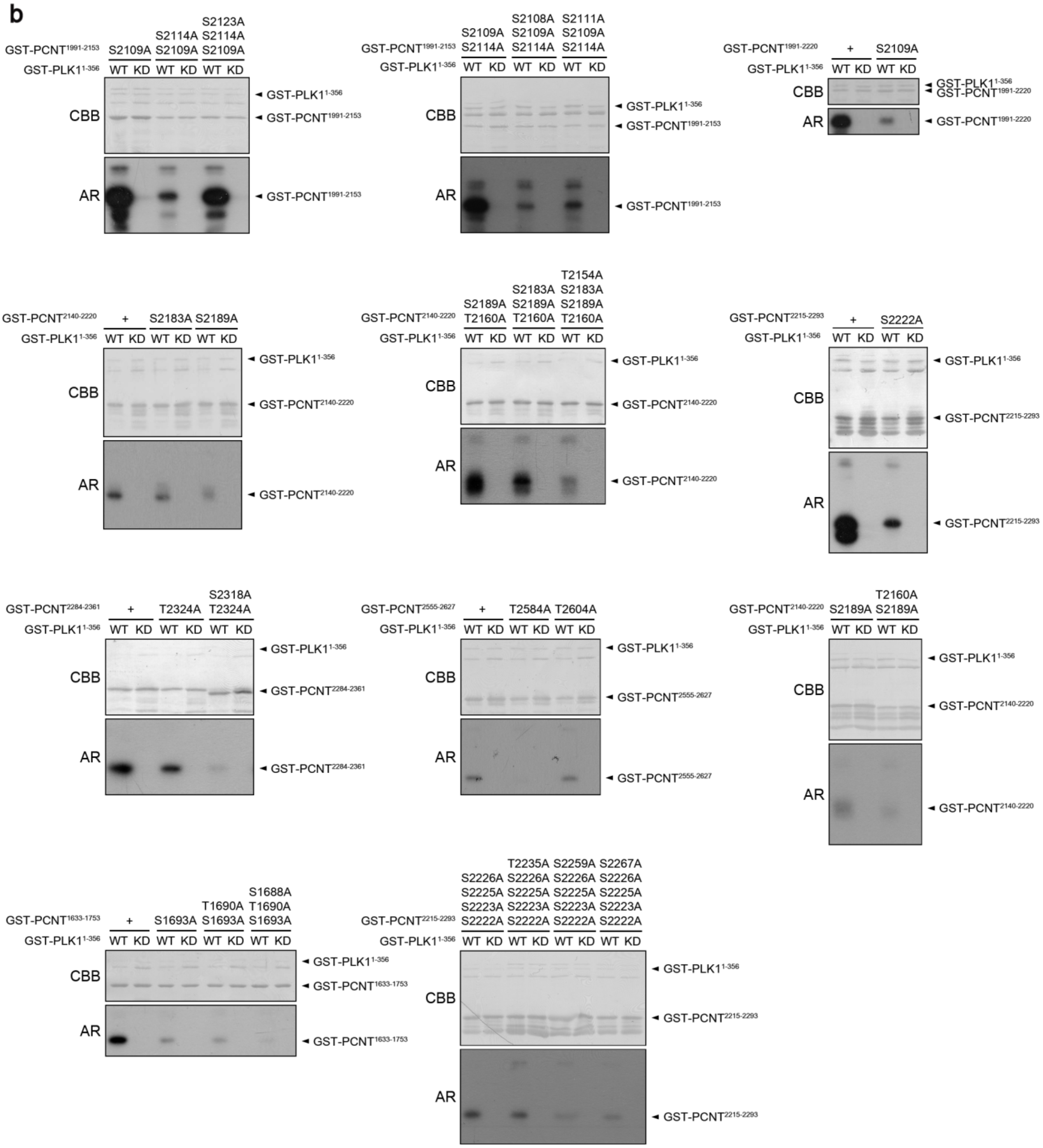

Supplementary Figure 1. PLK1 phosphorylation of PCNT *in vitro* (Cont'd)

(**a**,**b**) We performed *in vitro* kinase assays of PLK1 with PCNT fragments as substrates. The specific phosphorylation sites were confirmed with the PCNT fragments in which serine and threonine residues were substituted with alanines. In this way, we finally determined 22 residues for PLK1 phosphorylation. Results of T1209, T1221, S1229, S1235, and S1241 residues were previously described[10]. The phosphorylation sites of PCNT were grouped and colored with orange (T2584), yellow (AA; S1235A and S2141A), gray (7A; S1688A, T1690A, S1693, S2108, S2109, S2111, and S2114), green (9D or 9A; T2154A, T2160A, S2183A, S2189A, S2222A, S2259A, S2267A, S2318A, and T2324A), and dark green (2A; S2259A, S2267A). WT, wild type; KD, kinase dead.

Supplementary Figure 2. Centrosomal localization of the ectopic FLAG-PCNT-Myc proteins

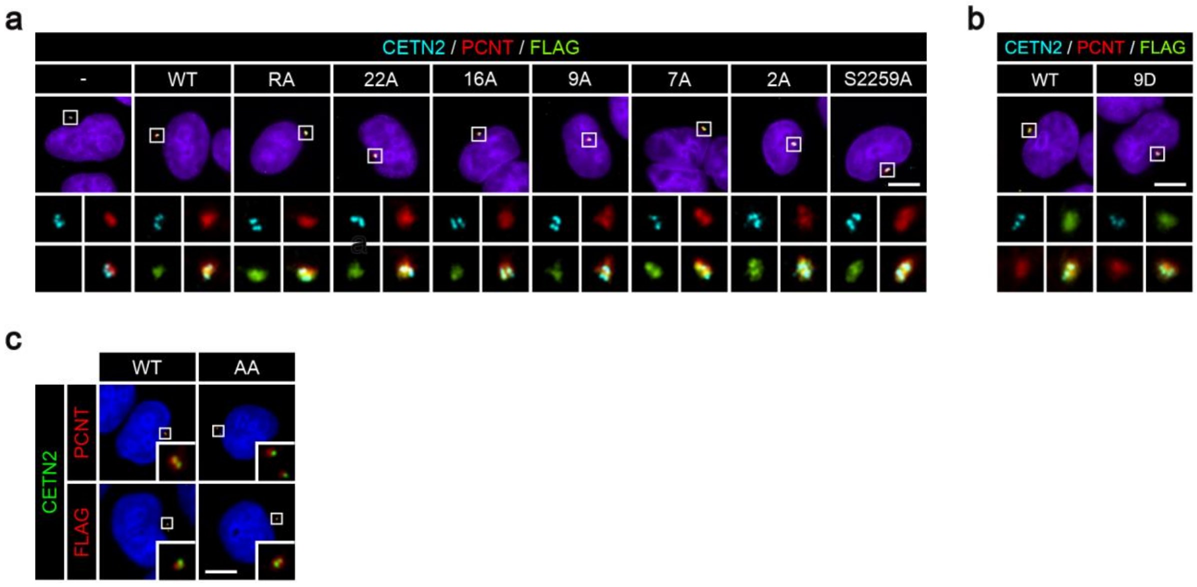

(**a**,**b**,**c**) The HeLa cells stably expressing FLAG-PCNT-Myc mutants were coimmunostained with antibodies specific to centrin-2 (CETN2; cyan in **a**,**b** and green in **c**), PCNT (red), and FLAG (green in **a**,**b** and red in **c**). DNA was visualized with DAPI (violet in **a**,**b** and blue in **c**). Scale bars, 10 μm

Supplementary Figure 3. Multiple phosphorylation sites for PCNT cleavage

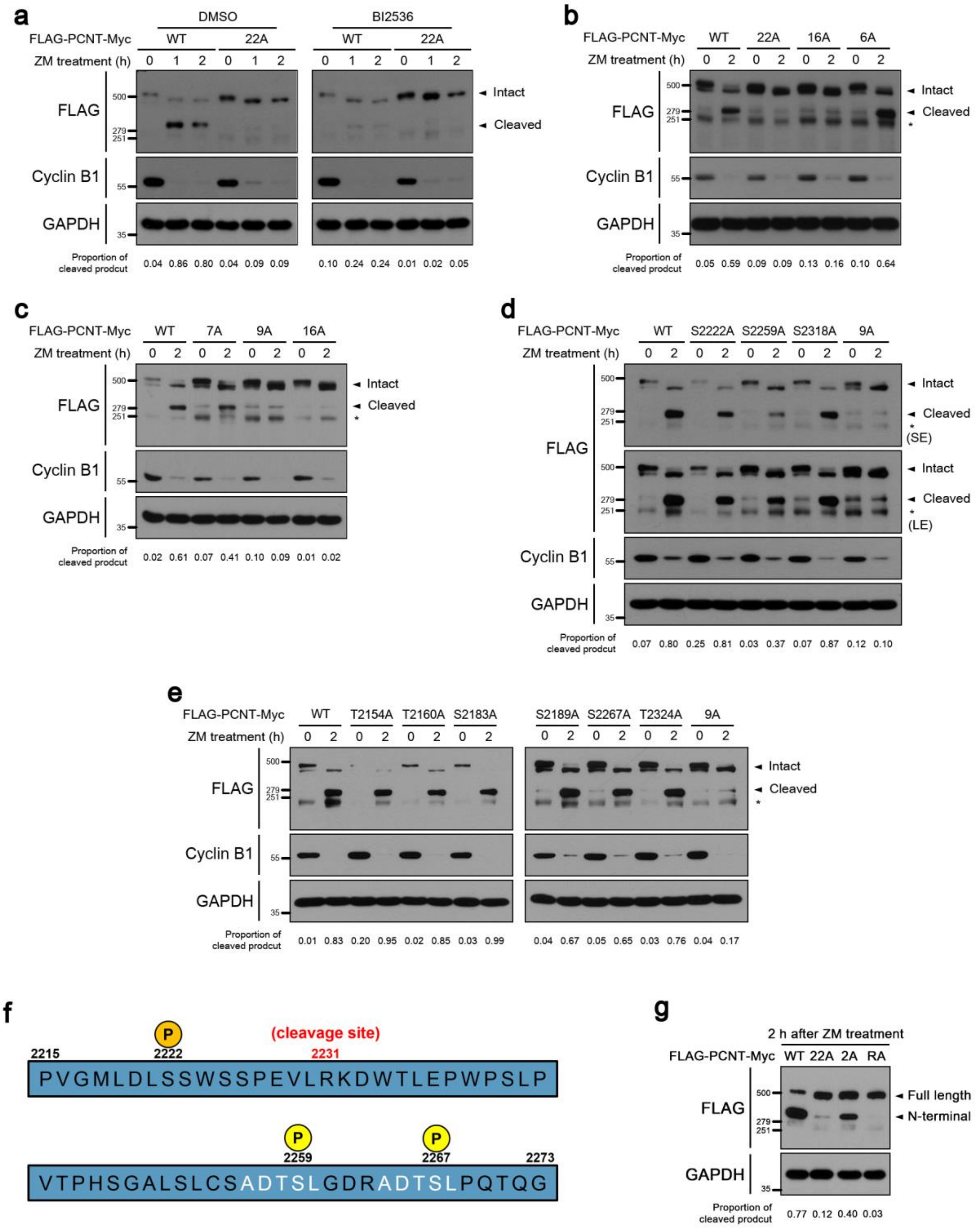

Supplementary Figure 3. Multiple phosphorylation sites for PCNT cleavage (Cont'd)

(**a**) The HeLa cells stably expressing FLAG-PCNT-Myc and FLAG-PCNT^{22A}-Myc were arrested at M phase and treated with BI2536 for 3 h. After the ZM447439 (ZM) treatment, the cells were harvested at indicated time points and subjected to immunoblot analyses with antibodies specific to FLAG, cyclin B1 and GAPDH. (**b-e**) The HeLa cells were transiently transfected with indicated FLAG-PCNT-Myc mutants and subjected to PCNT cleavage assays. (**f**) The amino acid sequences near the cleavage site (R2231) of PCNT. S2222, S2259, and S2267 are specifically phosphorylated by PLK1 *in vitro* (yellow). The ADT(pS)L sequence (white) is repeated twice. (**g**) Cleavage bands of ectopic PCNT mutants in the stable cell lines were determined during mitotic exit. All experiments were independently repeated at least twice.

Supplementary Figure 4. Centrosomal localization of the PCNT sensor proteins

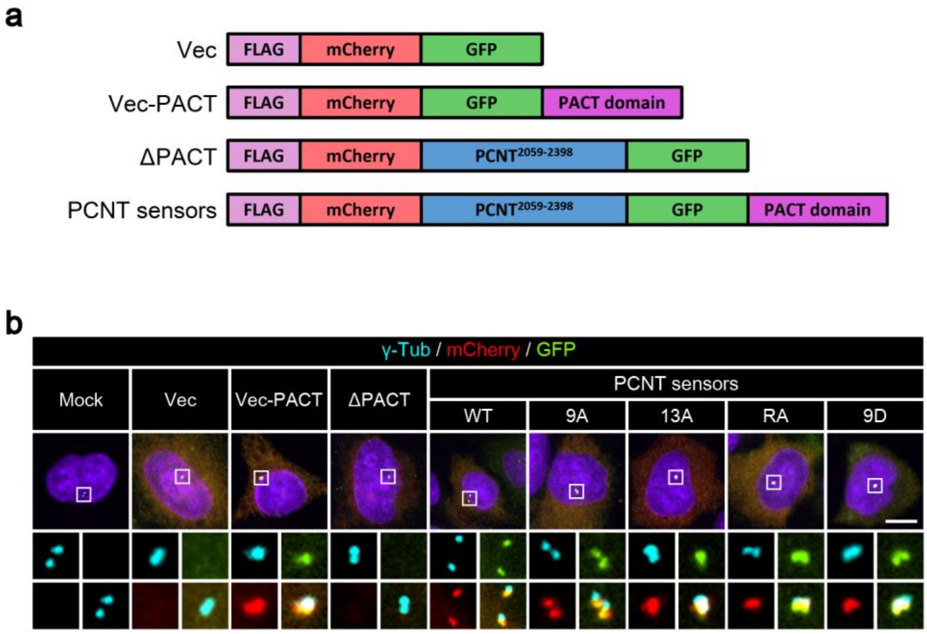

(**a**) Constructs of PCNT sensors (FLAG-mCherry-PCNT$^{2059\text{-}2398}$-GFP-PACT). mCherry (red) and GFP (green) were tagged at N- and C-terminus of PCNT$^{2059\text{-}2398}$ fragment (blue), respectively. PACT domain (violet) were attached at the end of GFP. FLAG (pink) was additionally tagged at N-terminus

of mCherry. Vec, Vector. (**b**) HeLa cells transiently transfected with the PCNT sensor proteins were coimmunostained with antibodies specific to γ-tubulin (γ-Tub, cyan), mCherry (red) and GFP (green). DNA were visualized with DAPI (violet). Scale bar, 10 μm.

Supplementary Figure 5. Characterization of the pS2259PCNT antibody

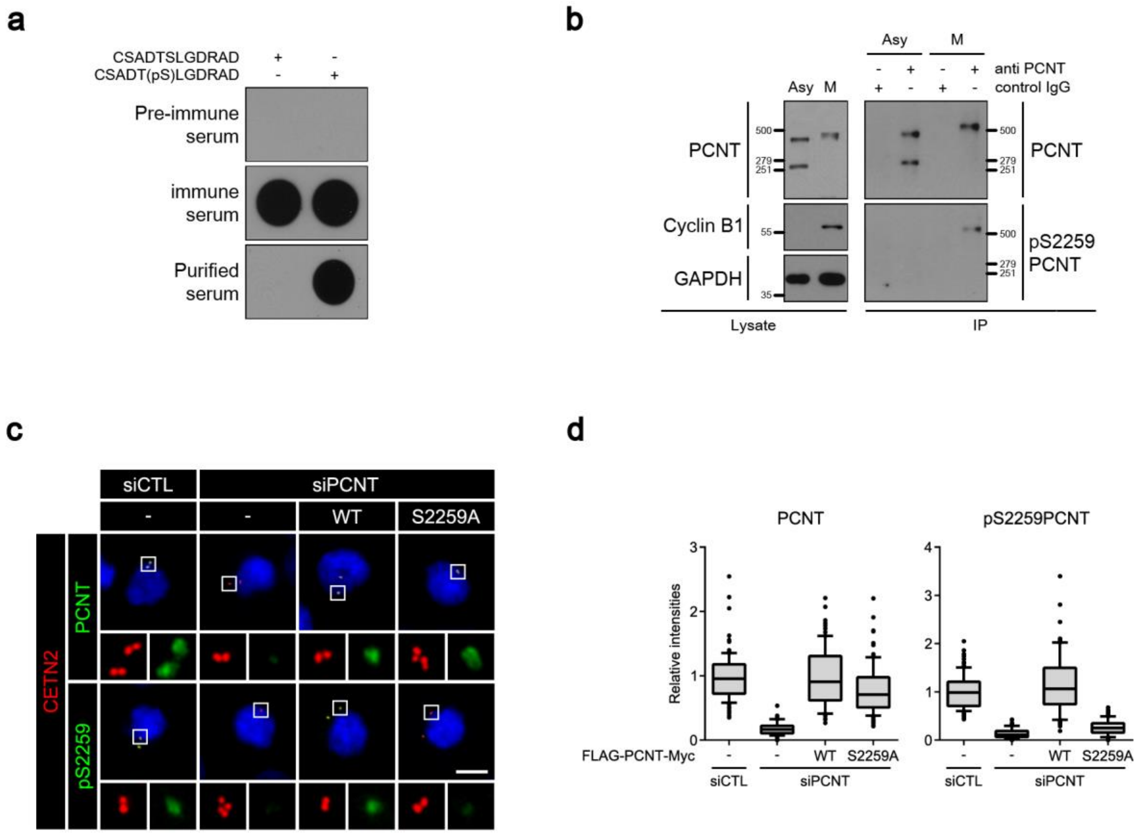

(**a**) The pS2259PCNT anti-serum was affinity-purified, and subjected to dot blot analyses with the synthetic peptides with or without pS2259. (**b**) Asynchronous and mitotic arrested lysates were immunoprecipitated with control IgG or the PCNT antibody and followed by immunoblot analysis with antibodies against PCNT and pS2259PCNT. (**c**) Endogenous PCNT was depleted in the cells stably expressing FLAG-PCNT-Myc (WT) or FLAG-PCNT^{S2259A}-Myc (S2259A). The cells were coimmunostained with the centrin-2 antibody (CETN2, red), along with the PCNT (red) and pS2259PCNT (red) antibodies. DNA was visualized with DAPI (blue). Scale bar, 10 μm (**d**) The centrosomal signals of PCNT and pS2259PCNT were determined and analyzed with the box-and-whisker plot. n=90 per group in 3 independent experiments.

Supplementary Figure 6. Generation of the FLAG-PCNT-Myc-rescued cells

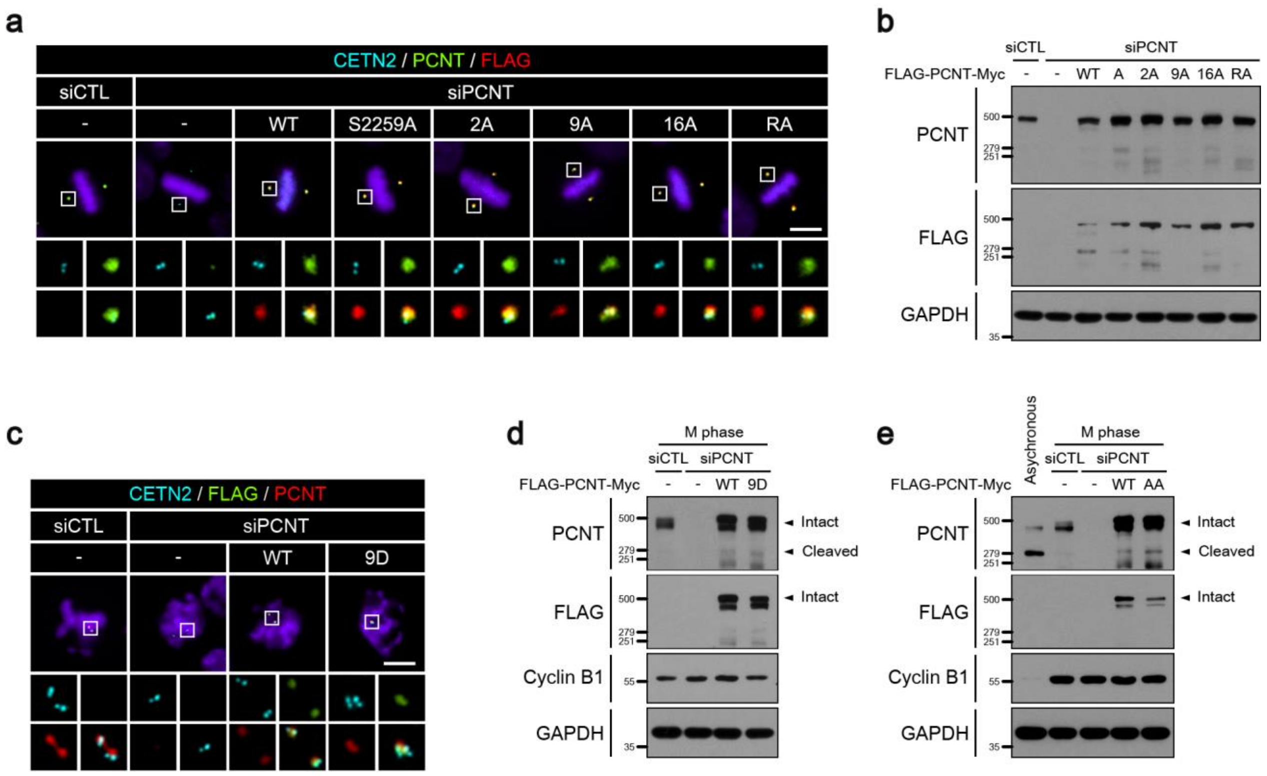

(**a**,**c**) Endogenous PCNT was depleted in HeLa cells in which the FLAG-PCNT-Myc mutant proteins were stably expressed. Mitotic cells were coimmunostained with antibodies against centrin-2 (CETN2, cyan), PCNT (green), and FLAG (red). DNA was visualized with DAPI (violet). Scale bars, 10 μm. (**b**) The PCNT rescued cells were arrested in G1/S phase with the double thymidine block method and subjected to immunoblot analyses with antibodies specific to PCNT, FLAG, and GAPDH. 'A' of lane 4 means FLAG-PCNT^{S2259A}-Myc. (**d,e**) The HeLa cells rescued with FLAG-PCNT^{9D}-Myc (9D) (**d**) and FLAG-PCNT$^{S2259/2267A}$-Myc (AA) (**e**) were arrested at M phase and subjected to immunoblot analysis with antibodies specific to PCNT, FLAG, cyclin B1, and GAPDH.

Supplementary Figure 7. Scans of X-ray films for immunoblot

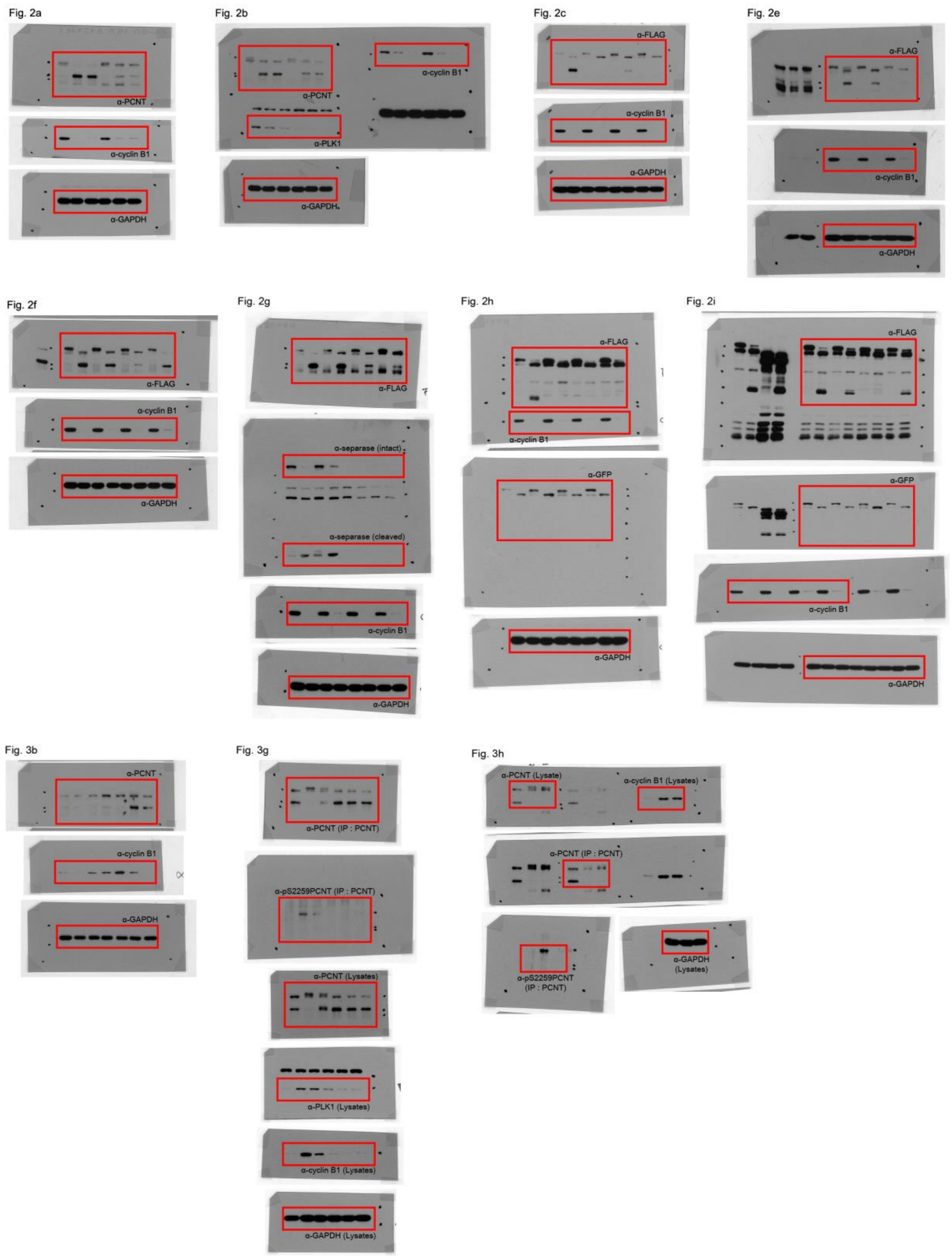

Supplementary Figure 7. Scans of X-ray films for immunoblot (Cont’d)

Fig. 5a

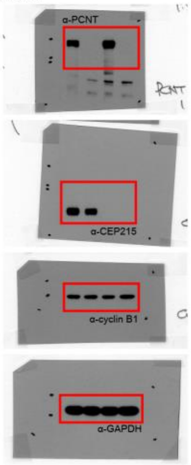

Fig. 7a

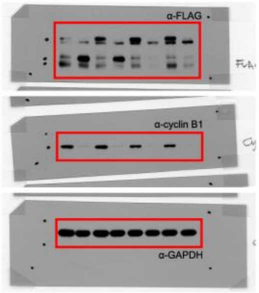

Supplementary Fig. 3a

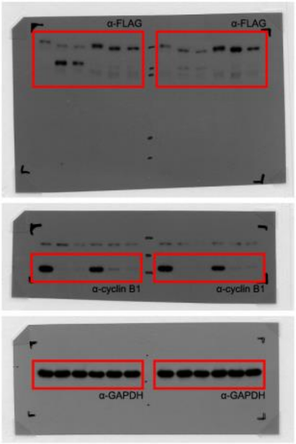

Supplementary Fig. 3b

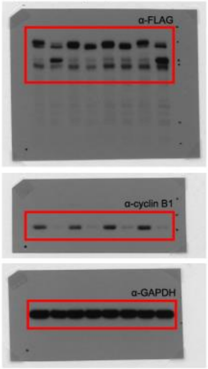

Supplementary Fig. 3c

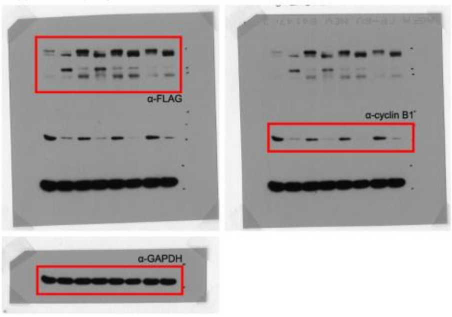

Supplementary Fig. 3d

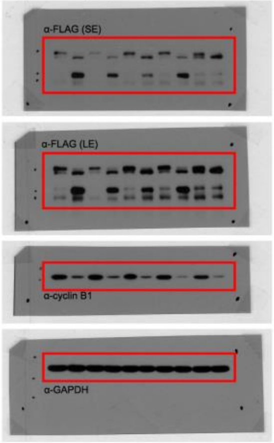

Supplementary Fig. 3e

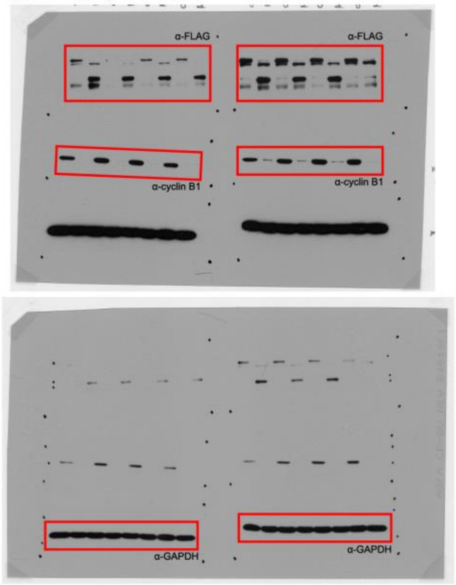

Supplementary Fig. 3g

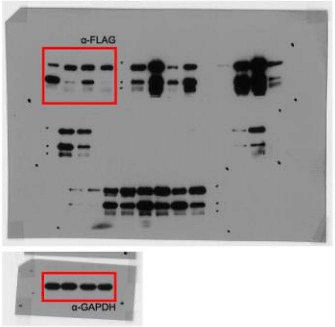

Supplementary Figure 7. Scans of X-ray films for immunoblot (Cont'd)

Supplementary Fig. S5a

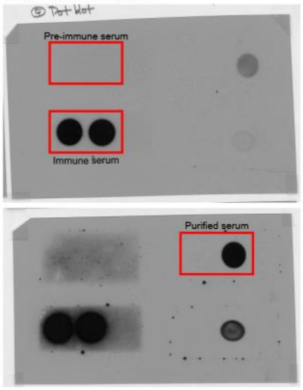

Supplementary Fig. S5b

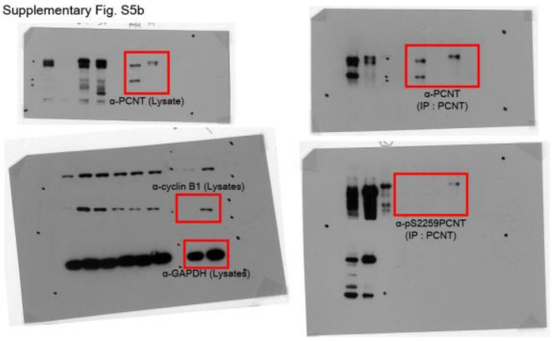

Supplementary Fig. S6b

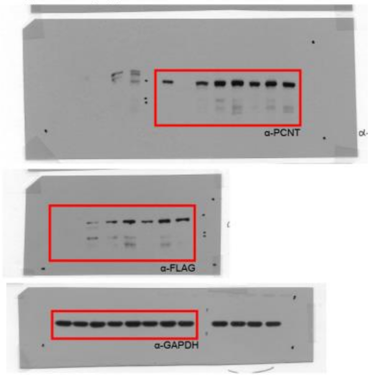

Supplementary Fig. S6d

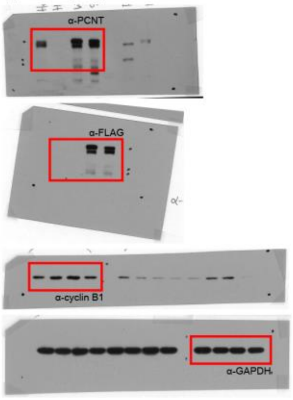

Supplementary Fig. S6e

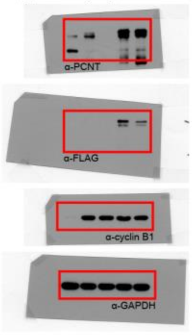

Supplement: Supplementary Information — Supplementary Figures 1-7 [file ncomms10076-s1.pdf]
